# The mediating effect of leisure activities in the relationship between depression and cognitive decline in middle age and older adults in Taiwan

**DOI:** 10.1186/s12877-023-03984-1

**Published:** 2023-05-22

**Authors:** Yu-Chan Hung, Wai-Lam Lao, Chih-Jung Yeh, Meng-Chih Lee

**Affiliations:** 1 Department of Public Health, Chung Shan Medical University, No.110, Sec.1, Jianguo N.Rd, Taichung City, 40201 Taiwan; 2 Department of Family Medicine, Taichung Hospital, Ministry of Health and Welfare, No. 199, Section 1, Sanmin Rd, West District, 403 Taichung City, Taiwan; 3 Institute of Medicine, Chung Shan Medical University, No.110, Sec.1, Jianguo N.Rd, Taichung City, 40201 Taiwan; 4 Institute of Population Health Sciences, National Health Research Institutes, No.35, Keyan Road, Zhunan Town, Miaoli County, 35053 Taiwan

**Keywords:** Depression, Cognitive function, Mediating effect, Intellectual-leisure activities, Mobility

## Abstract

**Background:**

Depression can affect the development of cognitive functions, and there are many people with depressive symptoms and cognitive decline in the aging population. The role of mediators between depressive symptoms and the subsequent cognitive decline remains unclear. We aimed to investigate whether depressive symptoms can slow down cognitive decline through a mediator.

**Methods:**

A total of 3,135 samples were collected in 2003, 2007, and 2011. This study used the CES-D10 and SPMSQ (Short Portable Mental State Questionnaire) to measure depression and cognitive functions. The effect of depression trajectory on the subsequent cognitive dysfunction was analyzed using multivariable logistic regression, and the mediating effect was analyzed using the Sobel test.

**Results:**

The results of the multivariable linear regression analysis showed that after including different variables in each model, such as leisure activities and mobility in 2003 and 2007, women had a higher percentage of depressive symptoms in each model, compared to men. The effect of depression in 2003 on cognitive decline in 2011 was mediated by intellectual leisure activities in 2007 in men (Z=-2.01) and physical activity limitation in 2007 in women (Z=-3.02).

**Conclusions:**

The mediation effect of this study shows that people with depressive symptoms will reduce their participation in leisure activities, which will lead to the degeneration of cognitive function. We suggest that if depressive symptoms are addressed as early as possible, people will have the ability and motivation to delay the decline of cognitive function through participation in leisure activities.

**Supplementary Information:**

The online version contains supplementary material available at 10.1186/s12877-023-03984-1.

## Introduction

Depression can affect cognitive functions, and there are many people with depressive symptoms and cognitive decline in the aging population [1, 2]. Taiwan became an aging society in 2018. The rapid growth of the older population will lead to a quick increase in the prevalence of chronic diseases and physical function degeneration, which may impact the entire society and economy [3].

Maintaining an active lifestyle is considered one of the keys to successful aging [4], leisure activities are associated with physical, mental, social and the overall health, while staying healthy can reduce mortality [5] and physical dysfunction [6] as well as increase perceived health [7].

Leisure activities can reduce the risk of cognitive decline (mental, physical and social activity) [8], depressive symptoms (intellectual, social, physical activity and gardening) [9], and mortality (social activities, more solitary activities and productive activities) [10], and increase happiness, cognitive functions, and psychological well-being (solitary, informal, and formal activities) [11]. In addition, leisure activities can be divided into four categories: intellectual, physical, social, and gardening, but only the first three have protective effects against depressive symptoms [9]. A longitudinal observational study (n = 15,582) that explored the risk of intellectual activities and dementia in the older adults. The study used multivariable logistic regression analysis to verify whether participating in intellectual activities at baseline was associated with a lower incidence of dementia and the results showed that intellectual leisure activities had a protective effect on dementia [12]. Another study showed that social activities can maintain mobility and reduce the risk of death because many social activities involve physical activity [13].

Mild cognitive impairment (MCI) is the stage between the expected decline in memory and thinking that happens with age and the more serious decline of dementia. The typical symptoms of MCI included: forget things more often, miss appointments or social events, have trouble following a conversation, find it hard to make decisions…etc. MCI and disability were once thought to be independent factors; however, they are now considered correlated. Slow gait speed can predict subsequent functional limitations or even disability in physical activities [14]. However, there is increasing evidence that cognitive functions play a more important role in slow gait speed [15]. Studies have found that slow gait speed and low grip strength can predict cognitive decline 10 years later [16].

The Korean study (used Korean Longitudinal Study of Aging (KLoSA) database, n = 4,374) aimed to identify the trajectory of depressive symptoms and to assess the relationship between depression trajectories and MMSE scores in the late middle-aged and older South Korean population. It was found that each trajectory group for depressive symptoms was associated with cognitive decline [17].

The study of Netherlands used Rotterdam Study (a population-based study) that has been conducted since 1990 and the samples was Rotterdam people who over 55 years old. This study used these data to identify depression trajectory and to study the subsequent risk of dementia. Different depression trajectories were associated with different dementia risk and the higher risk of dementia only in the increasing trajectory of depression [18].

Another study in Taiwan, used the Taiwan Longitudinal Study of Aging (TLSA) database from 1993 to 2007 (n = 2,300). This study used a group-based trajectory model to develop trajectories of cognitive decline (according to SPMSQ scores) and found depression score and other factors (physical activity, self-rated health, and cardiovascular diseases…etc.) were related to the cognitive impairment trajectories over time. Depression, physical function impairment, chronic diseases, and social capital might be potential risk factors of cognitive decline [19].

A study using the English Longitudinal Study of Ageing (ELSA) database conduct a study to investigate longitudinal association between the duration of depressive symptoms and subsequent cognitive decline (n = 7,610). During the 10-year follow-up, this study found depression were significantly associated with subsequent cognitive decline [20].

These studies have found that depression can affect the development of cognitive functions [17–20], and many people have depressive symptoms and cognitive function decline due to social aging [21, 22].

Treatments for depressive symptoms include pharmacologic treatment, electroconvulsive therapy (ECT), and psychosocial therapies [23]; cognitive decline can also be improved with medication, behavioral therapy, and psychotherapy [24]. However, the relationship between lifestyle factors, cognitive decline, and depressive symptoms has not been extensively studied. The role of mediators between depressive symptoms and the subsequent cognitive decline remains unclear.

Therefore, our aim in this study, we used lifestyle factors as mediators to explore the relationship between depression and cognitive decline.

We aim were (1) to determine whether cognitive decline in depressive patients can be slowed down through certain mediators, thereby reducing the prevalence of dementia, (2) used mediation analysis [25] to clarify whether leisure activities and mobility function limitations were mediators of depression and the subsequent cognitive decline.

## Methods

### Study sample

The Taiwan Longitudinal Study of Aging (TLSA) is a longitudinal survey with national representativeness in middle-aged and older people in Taiwan. Data were collected by the Health Promotion Bureau of the Ministry of Health and Welfare, Taiwan. Seven waves’ data were released at this time, in years 1989, 1993, 1996, 1999, 2003, 2007, and 2011. TLSA conducts three-stage stratified random sampling. In the first stage, the 331 townships and urban areas in Taiwan are divided into 27 layers according to the administrative area, education level and total fertility rate, and 56 townships and districts are randomly selected. In the second stage, neighborhoods were selected according to the proportion of the sample population in townships and districts. In the third stage, two older people were randomly selected from each neighborhood as sample cases. The samples in this study are all people over the age of 50. This study used 3 waves’ survey data from three waves: 2003, 2007, and 2011. In the fifth wave of survey in 2003, in addition to maintaining the original follow-up sample (the sample included in 1989 and in 1996), the middle-aged and older people aged 50–56 were selected as supplementary samples. The completion rate of follow-up visits for each generation was 79–91%, which is representative of the current situation of middle-aged and older people in Taiwan.

There were 5377 subjects in 2003, 4577 subjects in 2007, and 3727 subjects in 2011. To investigate the development of cognitive function decline, we excluded 266 subjects with abnormal cognitive function at baseline (year 2003). We excluded 376 subjects in 2003, 1,600 subjects in 2011, and none in 2007 as these subjects had missing values for cognitive function (due to death, loss of follow-up, or non-response). A total of 3,135 participants completed three waves of follow-up and provided complete data.

### Assessment of depression and cognitive function

#### CES-D10 (10-item centre for epidemiological studies depression scale)

We used the 10-item CES-D (10-item Centre for Epidemiological Studies Depression Scale (CES-D) [26] to assess depression, with the total score of 30, a maximum score of 3 for each item, and a minimum score of 0. The reliability of the CES-D questionnaire (standardized Cronbach’s alpha) was 0.83.

#### SPMSQ (short portable mental state questionnaire)

The SPMSQ was used to assess cognitive function [27], with a total score of 9. Participants with a total score < 6 were identified as having cognitive dysfunction. In this study, we excluded cases if their SPMSQ score < 6 in 2003 or did not respond to cognitive function in 2003 or in 2011. For the cases included in the study, we analyzed SPMSQ as a continuous variable.

### Assessment of other variables

The demographic variables (baseline, 2003) were age, gender, educational level and marital status. The lifestyle variables assessed included cigarette smoking and alcohol consumption.

Leisure activities are divided into intellectual, physical, social, and gardening; the first three have been found to have a positive effect on depression [9]. Intellectual leisure activities include watching TV, listening to the radio, reading newspapers/magazines/books/novels, playing chess or cards; physical leisure activities include walking, jogging, climbing, playing ball, and group activities such as singing, dancing, Tai Chi, Wai Dan Gong (one kind of qi gong, a system of deep breathing exercises) or singing karaoke; social leisure activities include chatting or brewing tea with relatives, friends, or neighbors; gardening leisure activities include planting flowers and gardening. Participants were investigated what leisure activities they like to do in the free time, and how often they did (less than once a month, 2–3 times a month, 1–2 times a week, almost every day).

There were eight questions in the mobility function assessment, with a total score of 24, and scores ranged from 0 to 3 (0 = no difficulty, 1 = a little difficult, 2 = very difficult, and 3 = cannot do it at all). The questions included the following: (1) standing continuously for about 15 min, (2) squatting, (3) raising both hands above the head, (4) holding or twisting things with fingers, (5) picking up or carrying 12 kg, (6) short-distance running (20–30 m), (7) walking for 200–300 m, and (8) climbing to the 2nd or 3rd floor. For reliability analysis, the standardized Cronbach’s alpha value was 0.85.

### Data analysis

#### Demographic analysis

Student’s t-test and chi-square test were applied for univariate analysis of the following variables: age, gender, education, marital status, cigarette smoking, alcohol drinking, mobility function, leisure activities, and depressive symptoms at baseline. The background characteristics of the samples used in this study were statistically significantly different from the excluded samples (shown in Appendix A).

#### Pearson correlation

Pearson’s correlation was applied to observe the relationship between cognitive decline (2011) and physical activity limitation, leisure activities, and depressive symptoms at baseline (2003) in males and females.

#### Multivariable linear regression analysis

We used multivariable linear regression to analyze the association between baseline depressive symptoms and cognitive function in 2011. Model 0 was adjusted for age, sex, marital status, education, smoking, and alcohol consumption at baseline. In addition to the adjusted variables in Model 0, total physical activities (2003, 2007), intellectual leisure activities (2003, 2007), physical leisure activities (2003, 2007), social leisure activities (2003, 2007), gardening leisure activities (2003, 2007), and mobility function (2003, 2007) were added to Models 1 to 6, respectively. The estimated reduction percentage (ERP) was calculated as ERP = [reg coeff_model1~6_ – reg coefficient _model 0_] / [0 – reg coefficient _model 0_]. We used variance inflation factor (VIF) method to test for multicollinearity.

#### Mediation analysis

**Fig. 1:**
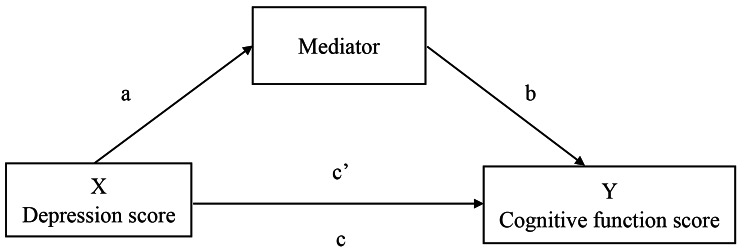
Illustration of the mediator analysis of depressive symptoms and cognitive functions

We performed a Sobel test with linear regression to present a, Sa, b, Sb, c, Sc, c, ‘and Sc’ and calculated the Z value with a, Sa, b, Sb. In Fig. 1, “a” represents the effect of the X variable on the mediator, “b” represents the effect of the mediator on the Y variable when the X variable is not considered, “c” represents the effect of the X variable on the Y variable when the mediator is not considered, and “c’” represents the effect of variable X on variable Y when the mediator is considered. In our study, the X variable represents the depression score, the Y variable represents the cognitive function score, and mediators include leisure activities and mobility function, while Sa, Sb, and Sc represent the standard errors of a, b, and c, respectively. The Bootstrap methods was also conduct in this study and the result was shown in Appendix B.

We used the Sobel test to analyze the mediation effects. If Z value is greater than 1.96 (the absolute value), this means that there was a significantly mediating effect [25, 28].

We identified the total physical activity, four types of leisure activities (intellectual, physical, social, and gardening). According the time sequence, depression may cause cognitive decline through some mediators, therefore we used mobility function in 2007 as mediators to examine the mediating effect of depressive symptoms (2003) on cognitive function decline (2011). All mediation variables at the baseline were controlled using the Sobel test.

## Results

The baseline characteristics of the participants are shown in Table 1. The average age of men (62.33 years) was higher than that of women (61.52 years), and the age over 75 years (15.1%) was also higher than that of women (11.8%). Regarding the educational level, 15.8% of men had a college degree or above, more than women (5.56%), and 13.82% of men and 30.35% of women had no spouse. In men, the percentages of cigarette smoking (41.46%) and alcohol drinking (51.91%) were higher compared to women’s cigarette smoking (2.88%) and alcohol drinking (15.85%).

**Table 1:** Summary characteristics of the participants at baseline (N = 3,135)

Variables	Female(n = 1565) Mean (SD)	Male(n = 1570) Mean (SD)	t/x^2^	p
**Age** ^**a,b**^	61.52 **±** 8.78	62.33 **±** 9.26	-2.51	0.0122
50-64yrs	1044(66.71)	984(62.68)	8.42	0.0148
65-75yrs	336(21.47)	349(22.23)		
>=75yrs	185(11.82)	237(15.10)		
**Educational level** ^**a**^			313.84	< 0.0001
Illiterate	387(24.73)	89(5.67)		
Primary education	793(50.67)	740(47.13)		
Junior/high school education	298(19.04)	493(31.4)		
College degree or above	87(5.56)	248(15.80)		
**Spouse** ^**a**^			124.50	< 0.0001
Yes	1,090(69.65)	1353(86.18)		
No	475(30.35)	217(13.82)		
**Cigarette Smoking** ^**a**^			675.73	< 0.0001
Yes	45(2.88)	651(41.46)		
No	1,520(97.12)	919(58.54)		
**Alcohol Drinking** ^**a**^			454.86	< 0.0001
Yes	248(15.85)	815(51.91)		
No	1317(84.15)	755(48.09)		
**PA** ^**b**^	1.66 **±** 1.33	1.68 **±** 1.35	-0.29	0.7723
**Dep-S** ^**b**^	4.75 **±** 5.28	3.28 **±** 4.42	8.49	< 0.0001
**Cog-S** ^**b**^	8.57 ± 0.74	8.77 ± 0.54	-8.85	< 0.0001
**MO** ^**b**^	2.28 **±** 3.83	1.10 **±** 2.88	9.81	< 0.0001
**Int-LA** ^**b**^	2.10 **±** 1.16	2.65 **±** 1.12	-13.55	< 0.0001
**Phy-LA** ^**b**^	0.97 **±** 0.82	1.02 **±** 0.83	-1.97	0.0485
**Soc-LA** ^**b**^	0.65 **±** 0.48	0.71 **±** 0.45	-4.02	< 0.0001
**Gar-LA** ^**b**^	0.38 **±** 0.49	0.34 **±** 0.47	2.49	0.0129

a: Chi square test; b: Two-sample t test; Dep-S = Depression scores; Cog-S = Cognitive functioning scores; PA = Total Physical Activity; Int-LA = Intellectual-Leisure Activities; Phy-LA = Physical-Leisure Activities; Soc-LA = Social-Leisure Activities; Gar-LA = Gardening-Leisure Activities; MO = Mobility function

In men, depression score (3.28), mobility function (1.10), and gardening leisure activities (0.34) were significantly lower than those of women’s depression score (4.75), mobility function (2.28), and gardening leisure activities (0.38). However, in men, intellectual (2.65), physical (1.02), and social leisure activities (0.71) were significantly higher than those of women’s intellectual (2.10), physical (0.97), and social leisure activities (0.65).

As shown in Table 2, in men, cognitive decline (2011) was significantly and positively correlated with intellectual and physical leisure activities (r = 0.17 and 0.1, respectively) but negatively correlated with mobility function and depressive symptoms (r = -0.20 and − 0.10, respectively). In females, cognitive decline (2011) was significantly and positively correlated with intellectual, physical, and gardening leisure activities (r = 0.30, 0.12, and 0.12, respectively) but negatively correlated with mobility function and depressive symptoms (r = -0.30 and − 0.14, respectively).

**Table 2:** Pearson Correlations analysis by gender (male: top right part; female: lower left part)

	Cog-S (2011)	Dep-S (2003)	PA (2003)	Int-LA (2003)	Phy-LA (2003)	Soc-LA (2003)	Gar-LA (2003)	MO (2003)
Cog-S (2011)	1	-0.10^***^	-0.0085	0.17^***^	0.10^***^	0.044	0.043	-0.20^***^
Dep-S (2003)	-0.14^***^	1	-0.090^**^	-0.18^***^	-0.16^***^	-0.11^***^	-0.11^***^	0.30^***^
PA (2003)	0.12^***^	-0.20^***^	1	0.13^***^	0.55^***^	0.023	0.16^***^	-0.041
Int-LA (2003)	0.30^***^	-0.20^***^	0.10^***^	1	0.26^***^	0.023	0.14^***^	-0.19^***^
Phy-LA (2003)	0.12^***^	-0.20^***^	0.53^***^	0.28^***^	1	0.11^***^	0.18^***^	-0.12^***^
Soc-LA (2003)	0.011	-0.11^***^	0.068^**^	0.077^**^	0.16^***^	1	0.13^***^	-0.12^***^
Gar-LA (2003)	0.12^***^	-0.10^***^	0.15^***^	0.24^***^	0.21^***^	0.14^***^	1	-0.094^**^
MO (2003)	-0.30^***^	0.34^***^	-0.0039	-0.22^***^	-0.15^***^	-0.047	-0.11^***^	1

Cog-S = Cognitive function scores; Dep-S = Depression scores; PA = Total Physical Activity; Int-LA = Intellectual-Leisure Activities; Phy-LA = Physical-Leisure Activities; Soc-LA = Social-Leisure Activities; Gar-LA = Gardening-Leisure Activities; MO = Mobility function; ***<0.0001; **<0.01; *<0.05

Table 3 shows the results of multivariable regression for depressive symptoms and the subsequent cognitive function decline. In Model 0, depressive symptoms were significantly associated with the subsequent cognitive decline in both the total sample (β=-0.021) and in females (β=-0.021). In Model 1 (Total Physical Activity), the Estimated Reduction Percentage (ERP) for depressive symptoms was 8.33% in men and 24.17% in women; in model 2 (Intellectual-Leisure Activities), the ERP was 28.14% in men and 40.06% in women, and there was a positive correlation between intellectual leisure activities in 2007 and cognitive decline (male = 0.12, p < 0.01, female = 0.13, p < 0.05); in model 3 (Physical-Leisure Activities), the ERP was 15.85% in men and 27.50% in women and, in men, the physical leisure activities in 2007 was positively correlated with cognitive decline (β = 0.088, p < 0.05); in model 4 (Social-Leisure Activities), the ERP was 6.10% in men and 18.17% in women; in model 5 (Gardening-Leisure Activities), the ERP was 1.69% in men and 33.28% in women and, in women, the gardening leisure activities in 2007 were positively correlated with the cognitive decline (β = 0.145, p < 0.05); in model 6 (Mobility function), the ERP was 71.95% in men but not available(NA) in women, and there was a negative correlation between mobility function in 2007 and cognitive decline in men and in women (β=-0.054 and − 0.044, respectively, p < 0.001). In most models, ERP values were higher in women. The detailed results for models 0–6 in Appendix C. After we used variance inflation factor (VIF) method to test for multicollinearity, there is not found multicollinearity from the analysis results.

**Table 3:** Multivariable regression of depressive symptoms and subsequent cognitive function decline

	Total subjects			Male			Female		
	β(ERP)	VIF		β(ERP)	VIF		β(ERP)	VIF	
Model 0: Dep-S(2003)	-0.012^**^	1.07		-0.012^‡^	1.04		-0.012^*^	1.07	
	R^2^ = 0.26, Adj. R^2^ = 0.25			R^2^ = 0.14, Adj. R^2^ = 0.13			R^2^ = 0.32, Adj. R^2^ = 0.32		
Model 1: Dep-S(2003)	-0.010(16.67%)	1.09		-0.011(8.33%)	1.05		-0.0091(24.17%)	1.09	
PA(2003)	-0.010	1.33		-0.0052	1.39		-0.018	1.29	
PA(2007)	0.040^*^	1.28		0.029	1.32		-0.040	1.25	
	R^2^ = 0.26, Adj. R^2^ = 0.26			R^2^ = 0.13, Adj. R^2^ = 0.13			R^2^ = 0.32, Adj. R^2^ = 0.32		
Model 2: Dep-S(2003)	-0.0080(33.33%)	1.09		-0.0085(28.14%)	1.06		-0.0070(40.06%)	1.09	
Int-LA(2003)	0.046^*^	1.65		0.038	1.48		0.043	1.69	
Int-LA(2007)	0.13^***^	1.44		0.12^**^	1.30		0.13^*^	1.49	
	R^2^ = 0.27, Adj. R^2^ = 0.27			R^2^ = 0.14, Adj. R^2^ = 0.14			R^2^ = 0.33, Adj. R^2^ = 0.32		
Model 3: Dep-S(2003)	-0.0096^*^(20.00%)	1.10		-0.0099(15.85%)	1.06		-0.0085 (27.50%)	1.09	
Phy-LA(2003)	0.018	1.37		0.027	1.36		-0.0023	1.38	
Phy-LA(2007)	0.084^**^	1.37		0.088^*^	1.36		0.088	1.38	
	R^2^ = 0.26, Adj. R2 = 0.26			R^2^ = 0.14, Adj. R^2^ = 0.13			R^2^ = 0.33, Adj. R^2^ = 0.32		
Model 4: Dep-S(2003)	-0.011^*^(8.33%)	1.09		-0.011(6.10%)	1.06		-0.0096(18.17%)	1.09	
Soc-LA(2003)	0.037	1.10		-0.0050	1.17		-0.035	1.04	
Soc-LA(2007)	0.065	1.07		0.10	1.12		0.043	1.06	
	R^2^ = 0.26, Adj. R^2^ = 0.26			R^2^ = 0.13, Adj. R^2^ = 0.13			R^2^ = 0.32, Adj. R^2^ = 0.32		
Model 5: Dep-S(2003)	-0.0099^*^(17.50%)	1.08		-0.012(1.69%)	1.05		-0.0079(33.28%)	1.08	
Gar-LA(2003)	0.037	1.20		-0.038	1.21		0.10	1.21	
Gar-LA(2007)	0.12^*^	1.21		0.073	1.20		0.145^*^	1.21	
	R^2^ = 0.26, Adj. R^2^ = 0.26			R^2^ = 0.13, Adj. R^2^ = 0.13			R^2^ = 0.33, Adj. R^2^ = 0.32		
Model 6: Dep-scores(2003)	0.00072(-)	1.19		-0.0033(71.95%)	1.13		0.0029(NA)	1.20	
Mo-function(2003)	-0.015	1.76		-0.0072	1.63		-0.016	1.77	
Mo-function(2007)	-0.051^***^	1.78		-0.054^***^	1.63		-0.044^***^	1.79	
	R^2^ = 0.29, Adj. R^2^ = 0.28			R^2^ = 0.16, Adj. R^2^ = 0.15			R^2^ = 0.34, Adj. R^2^ = 0.34		

Dep-S = Depression scores; PA = Total Physical Activity; Int-LA = Intellectual-Leisure Activities; Phy-LA = Physical-Leisure Activities; Soc-LA = Social-Leisure Activities; Gar-LA = Gardening-Leisure Activities; MO = Mobility function; all model adjusted for baseline age, education, spouse, cigarette smoking, alcohol drinking; ERP = Estimated Reduction Percentage; ***<0.0001; **<0.01; *<0.05; ‡=0.0513

**Fig. 2:**
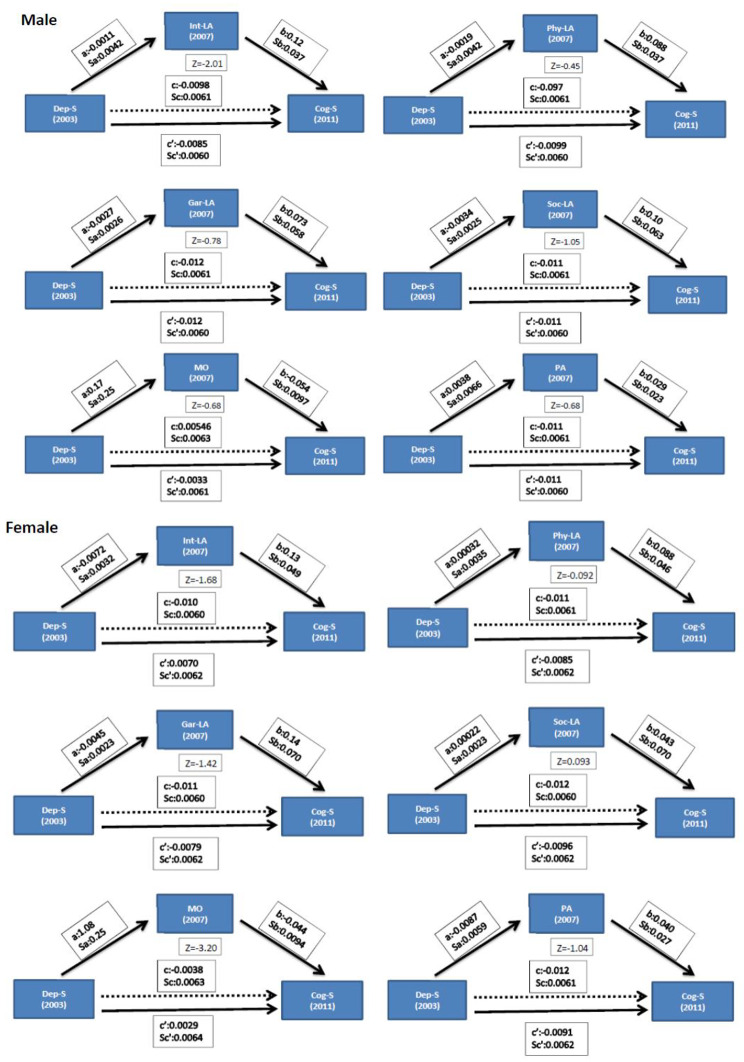
Depressive symptoms and the subsequent cognitive function decline with mediation analysis stratified by gender Note: Adjusted for age, education, spouse, cigarette smoking, alcohol drinking, and mediation variables at baseline

For both genders, mediation analysis for six mediators (Intellectual-Leisure Activities, Physical-Leisure Activities, Social-Leisure Activities, Gardening-Leisure Activities, Mobility Function, and Total Physical Activity) are shown in Fig. 2. In men, the estimated value of depressive symptoms in intellectual leisure activities was a=-0.0011, the standard error was Sa = 0.0042, and the estimated value of intellectual leisure activities in cognitive decline was b = 0.12 (Sb = 0.037). The Z-value of the mediating effect was − 2.01, indicating that the effect of depression in 2003 on cognitive decline in 2011 was significantly mediated by intellectual leisure activities in 2007. The mediation effects of the other five mediators were not statistically significant.

For women, the estimated value of depressive symptoms in mobility function was a = 1.08 (Sa = 0.25), and the estimated value of mobility function in cognitive decline was b=-0.044 (Sb = 0.0094). The Z value of the mediating effect was − 3.20, indicating that the effect of depression in 2003 on cognitive decline in 2011 was significantly mediated by mobility function in 2007. The mediation effects of the other five mediators were not statistically significant.

## Discussion

The results of our study are consistent with those of previous studies showing that physical or leisure activities have protective effects on mobility function, which, in turn, delays cognitive decline.

From the gender stratification analysis of the mediation effect, we confirmed that the mediator of depression symptoms (year 2003) and the subsequent cognitive decline (year 2011) were as follows: in men, the mediator was intellectual leisure activity in year 2007, and, in women, the mediator was mobility function in year 2007.

Some studies have found that leisure activities have a mediating effect on depressive symptoms and cognitive decline [29], while other studies have shown that older adults with depressive symptoms who participate in intellectual leisure activities have better cognitive functions [30]. Other studies have also found that disability is associated with depression and dementia [31–33].

In this study, the mediating effect between depressive symptoms and the subsequent cognitive decline in males was intellectual leisure activities. For middle-aged and older people with depressive symptoms, intellectual leisure activities are easier than other leisure activities because physical, social, and gardening activities require more physical effort, and middle-aged and older people with depressive symptoms are generally less active [34]. Therefore, the mediating effect of physical, social, and gardening leisure activities may be less obvious than that of intellectual leisure activities. Some studies indicated that, for men, intellectual leisure activities such as reading, listening to the radio, watching TV, card games, and other activities can reduce the risk of dementia [35]. Another study also found that intellectual leisure activities can reduce and delay the risk of dementia [12]. Intellectual leisure activities, such as chess or cards, that allow middle-aged and older people with depression symptoms to communicate with others, improve and maintain the flexibility and adaptability of the brain. It contributes to cognitive reserve [36, 37], thereby reducing cognitive decline. Therefore, choosing the appropriate leisure activity is better than blindly taking part in just any leisure activities.

This study found that improvement of mobility function was associated with a slowing of subsequent cognitive decline in women. Other studies have shown that women perform worse than men in activities such as housework, shopping and walking [38]. In this study, doing housework and shopping were linked to the hand muscle strength of the mobility function, whereas walking was linked to the foot muscle strength of the same function. Studies have indicated that slow gait is a precursor to subsequent cognitive decline [39] and that life-space mobility also affects the subsequent cognitive decline [40]. Limited living space mobility may reduce an individual’s social network, social integration, and social participation and is associated with cognitive decline in older adults [41].

Physical activity is known to enhance or maintain muscle mass and coordination of movements [42]. Studies have shown that long-term supplementation with multiple nutrients, such as vitamin D or a high-protein powder, with or without exercise, can increase muscle mass and strength in older adults with reduced mobility and sarcopenia [43, 44]. Regularly performing more than 48 min of moderate-intensity physical activity per week, such as walking, lower body strength training, flexibility, and balance training, can reduce the risk of disability [45]. In particular, lower body strength training can maintain muscle mass and reduce the risk of disability in the elderly [46]. In addition, participating in various types of leisure activities can also reduce the risk of subsequent disability, and people with early disability may not be willing to participate in subsequent leisure activities due to physical dysfunction [47].

There were significant differences in the background demographic characteristics of the included and excluded samples. We found that the samples not included in the study were usually older (n = 737, 46.06%) or less educated (primary education, n = 1,533, 48.90%), and these factors were associated with depression and cognitive decline. Our study may have underestimated, if included the samples that loss of follow-up, no response of cognitive questionnaire and dead in this study, the results may be more obvious.

### Limitations

The completion rate of this study in each wave was high, and the sample was taken from all middle-aged and older people in Taiwan; therefore, this study can represent middle-aged and older people in Taiwan. However, there are several limitations to this research: (1) participants who were assessed by the interviewer as unable to answer the depression or cognitive function questionnaires skipped this part of the questionnaire, so the subjects in this study were limited to healthy middle-aged and older people who could answer such questions; and (2) the use of self-report questionnaires may have recall bias. (3) In addition, this study used the Simple Mental State Questionnaire (SPMSQ), which can only screen out groups with mild cognitive impairment but cannot differentiate its severity.

## Conclusions

This 8-year long-term follow-up study confirmed the causal and mediating effects of depressive symptoms on the subsequent cognitive decline; in men, they are mediated through intellectual leisure activities and, in women, through mobility function. The mediation effect of this study shows that people with depressive symptoms will reduce their participation in leisure activities, which will lead to the degeneration of cognitive function. Therefore, we suggest that if depressive symptoms are addressed as early as possible, people will have the ability and motivation to delay the decline of cognitive function through participation in leisure activities.

The investigation of modifiable mediators in future research would shed light on appropriate and effective recommendations to prevent cognitive function decline in men and women, despite gender heterogeneity.

## Electronic supplementary material

Below is the link to the electronic supplementary material.

**Appendix A.** The statistical tests for background characteristics of the samples used in the study and the excluded samples (loss of follow-up, no response of cognitive questionnaire and dead).

**Appendix B.** The mediation effect analysis conduct by Bootstrap method (adjusted? baseline age, education, spouse, smoking, drinking and mediator).

**Appendix C.** The detailed results for models 0–6 of Table 3.

## Data Availability

The datasets generated during the current study are not publicly available, but data are however available from the applicants upon reasonable request and with permission of the Ministry of Health and Welfare in Taiwan. The data can be made available upon reasonable request from the Corresponding author.
