# Supplementary material for: The mediating effect of leisure activities in the relationship between depression and cognitive decline in middle age and older adults in Taiwan
Source: BMC Geriatr. 2023 May 22;23:315. doi: 10.1186/s12877-023-03984-1 (PMC10201706; doi:10.1186/s12877-023-03984-1)
Supplement: Supplementary file 1 — Appendix A. The statistical tests for background characteristics of the samples used in the study and the excluded samples (loss of follow-up, no response of cognitive questionnaire and dead). [file 12877_2023_3984_MOESM1_ESM.docx]

**Appendix A**

The statistical tests for background characteristics of the samples used in the study and the excluded samples (loss of follow-up, no response of cognitive questionnaire and dead).

|  | **Excluded samples**  **(n=1,061)** | **Included samples**  **(n=3,135)** | **x^2^** | **p** |
| --- | --- | --- | --- | --- |
|  | **n (%)** | **n (%)** |  |  |
| **Gender** |  |  |  |  |
| Male | 955(59.69%) | 1,570(50.08%) | 39.289 | <0.0001 |
| Female | 645(40.31%) | 1,565(49.92%) |  |  |
| **Age** |  |  |  |  |
| <65 | 490(30.63%) | 2,028(64.69%) | 692.16 | <0.0001 |
| 65-74 | 373(23.31%) | 685(21.85%) |  |  |
| >=75 | 737(46.06%) | 422(13.46%) |  |  |
| **Educational level** |  |  |  |  |
| Illiterate | 365(22.18%) | 476(15.18%) | 54.87 | <0.0001 |
| Primary education | 783(48.94%) | 1,533(48.90%) |  |  |
| Junior/high school education | 332(20.75%) | 791(25.63%) |  |  |
| College degree or above | 120(7.50%) | 335(10.69%) |  |  |
