# Supplementary material for: The mediating effect of leisure activities in the relationship between depression and cognitive decline in middle age and older adults in Taiwan
Source: BMC Geriatr. 2023 May 22;23:315. doi: 10.1186/s12877-023-03984-1 (PMC10201706; doi:10.1186/s12877-023-03984-1)
Supplement: Supplementary file 2 — Appendix B. The mediation effect analysis conduct by Bootstrap method (adjusted? baseline age, education, spouse, smoking, drinking and mediator). [file 12877_2023_3984_MOESM2_ESM.docx]

**Appendix B**

The mediation effect analysis conduct by Bootstrap method (adjusted：baseline age, education, spouse, smoking, drinking and mediator).

**Male**

**Female**

After we used Bootstrap methods (resampling 5,000 times), we found that only the effect of depression in 2003 on cognitive decline in 2011 was significantly mediated by intellectual leisure activities in 2007 in women.

After comparing the analysis results of these two methods, the result of the intellectual leisure activities in 2007 of women in Sobel test is very close to the borderline significance (z=-1.68, p=0.08) and the mediation effects of the other mediators in men or in women were not statistically significant.
