# Supplementary material for: The mediating effect of leisure activities in the relationship between depression and cognitive decline in middle age and older adults in Taiwan
Source: BMC Geriatr. 2023 May 22;23:315. doi: 10.1186/s12877-023-03984-1 (PMC10201706; doi:10.1186/s12877-023-03984-1)
Supplement: Supplementary file 3 — Appendix C. The detailed results for models 0–6 of Table 3. [file 12877_2023_3984_MOESM3_ESM.docx]

**Appendix C.**

The detailed results for models 0–6 of Table 3.

| **Model 0** | **Total subjects** | |  | **Male** | |  | **Female** | |
| --- | --- | --- | --- | --- | --- | --- | --- | --- |
|  | **β** | **VIF** |  | **β** | **VIF** |  | **β** | **VIF** |
| **Dep-scores (2003)** | -0.01** | 1.07 |  | -0.01 | 1.04 |  | -0.01* | 1.07 |
| **Age** (ref: <65) |  | |  |  | |  |  | |
| 65-74 | -0.57*** | 1.12 |  | -0.38*** | 1.11 |  | -0.78*** | 1.13 |
| >=75 | -1.05*** | 1.17 |  | -0.73*** | 1.17 |  | -1.48*** | 1.19 |
| **Gender** (ref: female) | 0.25*** | 1.61 |  |  |  |  |  |  |
| **Education**  (Ref: Illiterate) |  | |  |  | |  |  | |
| Primary education | 0.94*** | 2.37 |  | 0.60*** | 5.10 |  | 0.97*** | 1.61 |
| Junior/high school education | 1.22*** | 2.32 |  | 0.82*** | 4.71 |  | 1.33*** | 1.58 |
| College degree or above | 1.27*** | 1.80 |  | 0.89*** | 3.40 |  | 1.43*** | 1.25 |
| **Spouse** (ref: No) | 0.17*** | 1.13 |  | 0.32*** | 1.06 |  | 0.02 | 1.12 |
| **Smoking** (ref: No) | -0.08 | 1.35 |  | -0.04 | 1.08 |  | -0.15 | 1.04 |
| **Drinking** (ref: No) | -0.12* | 1.24 |  | -0.13* | 1.06 |  | -0.06 | 1.06 |
|  | R^2=^0.26  Adj. R^2^=0.25 | |  | R^2=^0.14  Adj. R^2^=0.13 | |  | R^2^=0.32  Adj. R^2^=0.32 | |
| Dep-scores=Depression scores; PA=Physical Activity; Int-LA= Intellectual-Leisure Activities; Phy-LA= Physical-Leisure Activities; Soc-LA= Social-Leisure Activities;  Gar-LA= Gardening-Leisure Activities; Mo-function= Mobility function;  Note: *<0.05; **<0.01; ***<0.001 | | | | | | | | |

| **Model 1** | **Total subjects** | |  | **Male** | |  | **Female** | |
| --- | --- | --- | --- | --- | --- | --- | --- | --- |
|  | **β** | **VIF** |  | **β** | **VIF** |  | **β** | **VIF** |
| **Dep-scores** **(2003)** | -0.01* | 1.09 |  | -0.01 | 1.05 |  | -0.01 | 1.09 |
| **PA (2007)** | 0.04* | 1.28 |  | 0.03 | 1.32 |  | 0.04 | 1.25 |
| **PA (2003)** | -0.01 | 1.33 |  | -0.01 | 1.39 |  | -0.02 | 1.29 |
| **Age** (ref: <65) |  | |  |  | |  |  | |
| 65-74 | -0.56*** | 1.16 |  | -0.36*** | 1.16 |  | -0.77*** | 1.16 |
| >=75 | -1.03*** | 1.22 |  | -0.71*** | 1.22 |  | -1.43*** | 1.23 |
| **Gender** (ref: female) | 0.26*** | 1.60 |  |  |  |  |  |  |
| **Education**  (ref: Illiterate) |  | |  |  | |  |  | |
| Primary education | 0.95*** | 2.39 |  | 0.55*** | 5.20 |  | 1.00*** | 1.62 |
| Junior/high school education | 1.23*** | 2.37 |  | 0.77*** | 4.85 |  | 1.35*** | 1.60 |
| College degree or above | 1.27*** | 1.83 |  | 0.82*** | 3.50 |  | 1.45*** | 1.27 |
| **Spouse** (ref: No) | 0.19*** | 1.14 |  | 0.31*** | 1.06 |  | 0.05 | 1.14 |
| **Smoking** (ref: No) | -0.08 | 1.35 |  | -0.03 | 1.09 |  | -0.21 | 1.04 |
| **Drinking** (ref: No) | -0.11* | 1.23 |  | -0.13* | 1.07 |  | -0.06 | 1.06 |
|  | R^2=^0.26  Adj. R^2^=0.26 | |  | R^2=^0.14  Adj. R^2^=0.13 | |  | R^2^=0.32  Adj. R^2^=0.32 | |
| Dep-scores=Depression scores; PA=Physical Activity; Int-LA= Intellectual-Leisure Activities; Phy-LA= Physical-Leisure Activities; Soc-LA= Social-Leisure Activities; Gar-LA= Gardening-Leisure Activities; Mo-function= Mobility function;  Note: *<0.05; **<0.01; ***<0.001 | | | | | | | | |

| **Model 2** | **Total subjects** | |  | **Male** | |  | **Female** | |
| --- | --- | --- | --- | --- | --- | --- | --- | --- |
|  | **β** | **VIF** |  | **β** | **VIF** |  | **β** | **VIF** |
| **Dep-scores (2003)** | -0.01 | 1.09 |  | -0.01 | 1.06 |  | -0.01 | 1.09 |
| **Int-LA (2007)** | 0.13*** | 1.45 |  | 0.12** | 1.30 |  | 0.13* | 1.49 |
| **Int-LA (2003)** | 0.05* | 1.65 |  | 0.04 | 1.47 |  | 0.04 | 1.69 |
| **Age** (ref: <65) |  | |  |  | |  |  | |
| 65-74 | -0.52*** | 1.13 |  | -0.33*** | 1.12 |  | -0.73*** | 1.16 |
| >=75 | -1.00*** | 1.18 |  | -0.69*** | 1.17 |  | -1.41*** | 1.21 |
| **Gender** (ref: female) | 0.22*** | 1.63 |  |  |  |  |  |  |
| **Education**  (ref: Illiterate) |  | |  |  | |  |  | |
| Primary education | 0.89*** | 2.52 |  | 0.48*** | 5.39 |  | 0.95*** | 1.71 |
| Junior/high school education | 1.08*** | 2.84 |  | 0.64*** | 5.34 |  | 1.19*** | 2.08 |
| College degree or above | 1.08*** | 2.16 |  | 0.67*** | 3.95 |  | 1.25*** | 1.50 |
| **Spouse** (ref: No) | 0.19*** | 1.14 |  | 0.32*** | 1.06 |  | 0.05 | 1.13 |
| **Smoking** (ref: No) | -0.06 | 1.36 |  | 0.00 | 1.11 |  | -0.23 | 1.04 |
| **Drinking** (ref: No) | -0.13** | 1.23 |  | -0.13* | 1.06 |  | -0.08 | 1.07 |
|  | R^2=^0.27  Adj. R^2^=0.27 | |  | R^2=^0.14  Adj. R^2^=0.14 | |  | R^2^=0.33  Adj. R^2^=0.32 | |
| Dep-scores=Depression scores; PA=Physical Activity; Int-LA= Intellectual-Leisure Activities; Phy-LA= Physical-Leisure Activities; Soc-LA= Social-Leisure Activities;  Gar-LA= Gardening-Leisure Activities; Mo-function= Mobility function;  Note: *<0.05; **<0.01; ***<0.001 | | | | | | | | |

| **Model 3** | **Total subjects** | |  | **Male** | |  | **Female** | |
| --- | --- | --- | --- | --- | --- | --- | --- | --- |
|  | **β** | **VIF** |  | **β** | **VIF** |  | **β** | **VIF** |
| **Dep-scores (2003)** | -0.01* | 1.10 |  | -0.01 | 1.06 |  | -0.01 | 1.09 |
| **Int-LA (2007)** | 0.08** | 1.37 |  | 0.09* | 1.36 |  | 0.09 | 1.38 |
| **Int-LA (2003)** | 0.02 | 1.37 |  | 0.03 | 1.36 |  | -0.002 | 1.38 |
| **Age** (ref: <65) |  | |  |  | |  |  | |
| 65-74 | -0.55*** | 1.12 |  | -0.35*** | 1.12 |  | -0.77*** | 1.14 |
| >=75 | -1.02*** | 1.18 |  | -0.70*** | 1.17 |  | -1.43*** | 1.20 |
| **Gender** (ref: female) | 0.26*** | 1.60 |  |  |  |  |  |  |
| **Education**  (ref: Illiterate) |  | |  |  | |  |  | |
| Primary education | 0.94*** | 2.41 |  | 0.53*** | 5.21 |  | 1.00*** | 1.62 |
| Junior/high school education | 1.20*** | 2.45 |  | 0.73*** | 4.92 |  | 1.32*** | 1.68 |
| College degree or above | 1.21*** | 1.92 |  | 0.75*** | 3.61 |  | 1.39*** | 1.31 |
| **Spouse** (ref: No) | 0.19*** | 1.14 |  | 0.32*** | 1.06 |  | 0.05 | 1.13 |
| **Smoking** (ref: No) | -0.07 | 1.36 |  | -0.01 | 1.11 |  | -0.20 | 1.04 |
| **Drinking** (ref: No) | -0.12* | 1.23 |  | -0.13* | 1.06 |  | -0.06 | 1.06 |
|  | R^2=^0.26  Adj. R^2^=0.26 | |  | R^2=^0.14  Adj. R^2^=0.13 | |  | R^2^=0.33  Adj. R^2^=0.32 | |
| Dep-scores=Depression scores; PA=Physical Activity; Int-LA= Intellectual-Leisure Activities; Phy-LA= Physical-Leisure Activities; Soc-LA= Social-Leisure Activities;  Gar-LA= Gardening-Leisure Activities; Mo-function= Mobility function;  Note: *<0.05; **<0.01; ***<0.001 | | | | | | | | |

| **Model 4** | **Total subjects** | |  | **Male** | |  | **Female** | |
| --- | --- | --- | --- | --- | --- | --- | --- | --- |
|  | **β** | **VIF** |  | **β** | **VIF** |  | **β** | **VIF** |
| **Dep-scores (2003)** | -0.01* | 1.09 |  | -0.01 | 1.06 |  | -0.01 | 1.08 |
| **Int-LA (2007)** | 0.06 | 1.07 |  | 0.10 | 1.12 |  | 0.04 | 1.04 |
| **Int-LA (2003)** | -0.04 | 1.10 |  | -0.01 | 1.17 |  | -0.04 | 1.06 |
| **Age** (ref: <65) |  | |  |  | |  |  | |
| 65-74 | -0.55*** | 1.12 |  | -0.34*** | 1.12 |  | -0.77*** | 1.14 |
| >=75 | -1.02*** | 1.19 |  | -0.69*** | 1.20 |  | -1.43*** | 1.20 |
| **Gender** (ref: female) | 0.26*** | 1.60 |  |  |  |  |  |  |
| **Education**  (ref: Illiterate) |  | |  |  | |  |  | |
| Primary education | 0.96*** | 2.39 |  | 0.55*** | 5.20 |  | 1.01*** | 1.61 |
| Junior/high school education | 1.24*** | 2.35 |  | 0.78*** | 4.84 |  | 1.37*** | 1.59 |
| College degree or above | 1.28*** | 1.81 |  | 0.84*** | 3.49 |  | 1.46*** | 1.25 |
| **Spouse** (ref: No) | 0.19*** | 1.13 |  | 0.31*** | 1.06 |  | 0.05 | 1.13 |
| **Smoking** (ref: No) | -0.09 | 1.35 |  | -0.04 | 1.09 |  | -0.21 | 1.04 |
| **Drinking** (ref: No) | -0.11* | 1.24 |  | -0.13* | 1.08 |  | -0.05 | 1.06 |
|  | R^2=^0.26  Adj. R^2^=0.26 | |  | R^2^=0.13  Adj. R^2^=0.13 | |  | R^2^=0.32  Adj. R^2^=0.32 | |
| Dep-scores=Depression scores; PA=Physical Activity; Int-LA= Intellectual-Leisure Activities; Phy-LA= Physical-Leisure Activities; Soc-LA= Social-Leisure Activities;  Gar-LA= Gardening-Leisure Activities; Mo-function= Mobility function;  Note: *<0.05; **<0.01; ***<0.001 | | | | | | | | |

| **Model 5** | **Total subjects** | |  | **Male** | |  | **Female** | |
| --- | --- | --- | --- | --- | --- | --- | --- | --- |
|  | **β** | **VIF** |  | **β** | **VIF** |  | **β** | **VIF** |
| **Dep-scores (2003)** | -0.01* | 1.08 |  | -0.01 | 1.05 |  | -0.01 | 1.08 |
| **Int-LA (2007)** | 0.12** | 1.21 |  | 0.07 | 1.20 |  | 0.14* | 1.21 |
| **Int-LA (2003)** | 0.04 | 1.20 |  | -0.04 | 1.21 |  | 0.10 | 1.21 |
| **Age** (ref: <65) |  | |  |  | |  |  | |
| 65-74 | -0.55*** | 1.12 |  | -0.35*** | 1.11 |  | -0.77*** | 1.14 |
| >=75 | -1.02*** | 1.18 |  | -0.70*** | 1.17 |  | -1.42*** | 1.20 |
| **Gender** (ref: female) | 0.27*** | 1.62 |  |  |  |  |  |  |
| **Education**  (ref: Illiterate) |  | |  |  | |  |  | |
| Primary education | 0.94*** | 2.40 |  | 0.55*** | 5.21 |  | 0.98*** | 1.63 |
| Junior/high school education | 1.21*** | 2.39 |  | 0.77*** | 4.85 |  | 1.31*** | 1.64 |
| College degree or above | 1.23*** | 1.87 |  | 0.82*** | 3.53 |  | 1.38*** | 1.29 |
| **Spouse** (ref: No) | 0.18*** | 1.14 |  | 0.31*** | 1.07 |  | 0.05 | 1.13 |
| **Smoking** (ref: No) | -0.08 | 1.35 |  | -0.03 | 1.08 |  | -0.21 | 1.04 |
| **Drinking** (ref: No) | -0.12* | 1.23 |  | -0.13* | 1.06 |  | -0.06 | 1.06 |
|  | R^2=^0.26  Adj. R^2^=0.26 | |  | R^2=^0.13  Adj. R^2^=0.13 | |  | R^2^=0.33  Adj. R^2^=0.32 | |
| Dep-scores=Depression scores; PA=Physical Activity; Int-LA= Intellectual-Leisure Activities; Phy-LA= Physical-Leisure Activities; Soc-LA= Social-Leisure Activities;  Gar-LA= Gardening-Leisure Activities; Mo-function= Mobility function;  Note: *<0.05; **<0.01; ***<0.001 | | | | | | | | |

| Model 6 | **Total subjects** | |  | **Male** | |  | **Female** | |
| --- | --- | --- | --- | --- | --- | --- | --- | --- |
|  | **β** | **VIF** |  | **β** | **VIF** |  | **β** | **VIF** |
| **Dep-scores (2003)** | 0.0007 | 1.19 |  | -0.003 | 1.13 |  | 0.003 | 1.20 |
| **Int-LA (2007)** | -0.05*** | 1.78 |  | -0.05*** | 1.63 |  | -0.04*** | 1.79 |
| **Int-LA (2003)** | -0.01 | 1.76 |  | -0.01 | 1.66 |  | -0.02 | 1.77 |
| **Age** (ref: <65) |  | |  |  | |  |  | |
| 65-74 | -0.42*** | 1.19 |  | -0.28*** | 1.14 |  | -0.60*** | 1.27 |
| >=75 | -0.81*** | 1.31 |  | -0.56*** | 1.26 |  | -1.18*** | 1.39 |
| **Gender** (ref: female) | 0.20*** | 1.63 |  |  |  |  |  |  |
| **Education**  (ref: Illiterate) |  | |  |  | |  |  | |
| Primary education | 0.91*** | 2.40 |  | 0.48*** | 5.25 |  | 0.98*** | 1.62 |
| Junior/high school education | 1.17*** | 2.38 |  | 0.71*** | 4.89 |  | 1.29*** | 1.60 |
| College degree or above | 1.20*** | 1.83 |  | 0.74*** | 3.53 |  | 1.39*** | 1.26 |
| **Spouse** (ref: No) | 0.18*** | 1.14 |  | 0.30*** | 1.06 |  | 0.05 | 1.13 |
| **Smoking** (ref: No) | -0.09 | 1.34 |  | -0.04 | 1.08 |  | -0.21 | 1.04 |
| **Drinking** (ref: No) | -0.14** | 1.23 |  | -0.16** | 1.07 |  | -0.05 | 1.06 |
|  | R^2=^0.29  Adj. R^2^=0.28 | |  | R^2=^0.16  Adj. R^2^=0.15 | |  | R^2^=0.34  Adj. R^2^=0.34 | |
| Dep-scores=Depression scores; PA=Physical Activity; Int-LA= Intellectual-Leisure Activities; Phy-LA= Physical-Leisure Activities; Soc-LA= Social-Leisure Activities; Gar-LA= Gardening-Leisure Activities; Mo-function= Mobility function;  Note: *<0.05; **<0.01; ***<0.001 | | | | | | | | |
